# The Psychological Burden of Surgery During a Pandemic: Evaluating Preoperative Anxiety in the COVID-19 Era

**DOI:** 10.7759/cureus.65466

**Published:** 2024-07-26

**Authors:** Avinash Prakash, Jyoti Baghel, Amrusha M Raipure, Prakash G Gondode, Omshubham G Asai, Bhuvaneswari Balasubramanian, Anita Yadav

**Affiliations:** 1 Department of Anaesthesiology and Critical Care, All India Institute of Medical Sciences, Nagpur, IND; 2 Department of Obstetrics and Gynaecology, Shri Ram Murti Smarak (SRMS) Institute of Medical Sciences, Bareilly, IND; 3 Department of Anaesthesiology and Critical Care, All India Institute of Medical Sciences, Delhi, IND; 4 Department of Obstetrics and Gynaecology, AIl India Institute of Medical Sciences, Nagpur, IND

**Keywords:** healthcare stressors, elective surgery, psychological impact, anaesthesia anxiety, surgery-related fears, covid-19 pandemic, preoperative anxiety

## Abstract

Background

The COVID-19 pandemic has introduced unprecedented challenges to global healthcare systems, including heightened psychological stress among patients. This study evaluates the preoperative anxiety levels among patients scheduled for surgery during the COVID-19 pandemic.

Methods

This cross-sectional observational study was conducted between April 2020 and March 2022. Adult patients aged 18-80 years, scheduled for elective or emergency surgery, were included. Exclusion criteria were mental illness, impaired communication, and hemodynamic instability. A pre-validated questionnaire addressing demographics, prior surgery exposure, surgery-related anxiety, and COVID-19-related fears was administered. Anxiety levels were scored on a 1-5 Likert scale. Data were analyzed using SPSS version 22 (IBM Corp., Armonk, USA).

Results

A total of 112 patients participated, with a mean age of 42.3±14.2 years. The majority were female (61 patients, 54.5%), married (96 patients, 85.7%), and resided in urban areas (85 patients, 75.9%). Most patients had no prior surgical history (87 patients, 77.7%). Surgery-related fears were prevalent, with 110 patients (98.2%) fearing surgical complications and 111 patients (99.1%) fearing postoperative pain. COVID-19-related fears were also significant, with 108 patients (96.4%) fearing infection during hospital stay and 100 patients (89.3%) fearing infecting family members. Mild fear was the most common anxiety level (70 patients, 62.95%), followed by moderate fear (25 patients, 22.5%).

Discussion

The study highlights the dual stressors of surgery and the pandemic, contributing to heightened preoperative anxiety. Findings indicate that significant anxiety levels were present, driven by fears related to surgery, anesthesia, and COVID-19. This aligns with other studies that report high preoperative anxiety levels exacerbated by the pandemic. The comprehensive assessment of anxiety factors underscores the need for tailored interventions to mitigate these anxieties.

Conclusion

The COVID-19 pandemic has significantly increased preoperative anxiety among surgical patients. Addressing both surgical and pandemic-related anxieties is crucial for improving patient outcomes. Healthcare providers should implement psychological support programs to alleviate these anxieties. Understanding the multifaceted nature of preoperative anxiety during the pandemic can enhance patient care.

## Introduction

The novel coronavirus 2019-nCoV (COVID-19) infection is a public health emergency of international concern. It was first detected in Wuhan, China, spreading rapidly to other countries worldwide. On the 12th of March 2020, the World Health Organization (WHO) announced the new coronavirus outbreak pandemic and according to WHO's official website, more than fifteen million people have been confirmed to have a COVID-19 infection globally [1]. The outbreak and diffusion of the COVID-19 infection have caused an emergency status in the health system. The pandemic has affected people of all nations, continents, races, and socioeconomic groups [2]. COVID-19 is characterized by fever (89%), cough (58%), dyspnea (46%), myalgias (29%), lymphopenia, and typical chest imaging features of bilateral ground glass opacities and consolidation. Although symptoms can range from mild to severe, 20% of infected patients overall require admission to an intensive care unit. Risk factors for severe disease or death include older age, smoking, chronic obstructive pulmonary disease, diabetes, hypertension, immune-compromised, and malignancy [3]. The fatality has till now been reported to be lower than the previous outbreaks of ebola, Severe Acute Respiratory Syndrome (SARS), and Middle East Respiratory Syndrome (MERS) [4].

The rapidly spreading outbreak imposes an unprecedented burden on the effectiveness and sustainability of our healthcare system. After the WHO declaration, elective surgeries at hospitals were cancelled due to the concern that elective procedures may contribute to the spreading of the coronavirus within facilities and use up medical resources needed to manage a potential surge of coronavirus cases. Arguably, the potential fallout from inconsiderate elective surgery cancellations may have a more dramatic and immeasurable impact on the health of our communities than the morbidity and mortality inflicted by the novel coronavirus disease [5]. The uncertainty on the predicted time course of COVID-19 beyond a critical inflection point implies that patients may be deprived of access to timely surgical care likely for many months to come.

The first and foremost responses to the pandemic have been fear and a sense of clear and imminent danger. During a pandemic, normal people are being exposed to extraordinary situations. Fear and anxiety about a new disease and what could happen can be overwhelming. The presentations are myriad and include emotional difficulties like anxiety, depression, sleep, and appetite disturbances. Pandemics pose a challenge to psychological resilience and can lead to heightened levels of stress due to fear [6]. Fear is an adaptive emotion that serves to mobilize the energy to deal with potential threats. When fear is too excessive, this may have detrimental effects on the health of an individual. It is a subjective conscious experience involving idiosyncratic concerns and fluctuations over time [7]. Fear and anxiety about a disease can lead to social stigma, which is negative attitudes toward people.

Preoperative anxiety among patients undergoing surgery under anesthesia is not an uncommon behavior. It is described as a vague, uneasy feeling in which the exact causes are often nonspecific and unknown to the individual but known to cause the body to react with undesirable haemodynamics because of sympathetic, parasympathetic, and endocrine stimulation. Surgery-related anxiety is somewhat commonly accepted as a normal reaction in preoperative patients. The literature reports that 60%-92% of patients experience significant preoperative anxiety [8]. Preoperative periods are worrying events that generate specific emotional, cognitive, and psychological responses in a patient. Autonomic response associated with increased anxiety may cause tachycardia, hypertension, and arrhythmias and increase the risk of intra-operative hypothermia [9]. Studies have shown that high preoperative anxiety levels can lead to an increased postoperative analgesic requirement, prolonged hospital stay, significant contribution to adverse peri-operative outcomes, and poor patient satisfaction [10,11].

We, therefore, hypothesized that the COVID-19 pandemic could have had a profound impact on the level of anxiety among the patients attending pre-anesthetic clinics which may be different according to their base level of anxiety. The aim of the present study is to assess preoperative anxiety among patients scheduled for elective/emergency surgery attending pre-anesthetic checkup (PAC) clinics during the COVID-19 pandemic.

## Materials and methods

This cross-sectional observational study was conducted amongst the patients posted for elective/ emergency surgery at a tertiary care hospital in central India from April 2020 to March 2022. The study included adults within the age group of 18-80 years with or without a history of previous surgery who were posted for surgery. Patients with mental illness, impaired communication, and hemodynamically unstable were excluded from the study. All willing patients provided written informed consent to participate in the study. They were assured of complete confidentiality and anonymity of the data. The Institutional Ethics Committee of the All India Institute of Medical Sciences, Nagpur approved the study (approval number: IEC/Pharmac/2020/150). The questionnaire was submitted to five experts comprising four professionals in the field of Anaesthesia and one statistician for content validity and was pretested over 10 patients who attended the pre-anaesthesia check-up (PAC) clinic. A simple questionnaire, modified from validated questionnaires, was prepared after reviewing the bibliography and after expert consultation. The modified questionnaire underwent content validation by five experts (four professionals in the field of anaesthesia and one statistician) and was pretested on 10 patients who attended the pre-anaesthesia check-up (PAC) clinic. This pre-validated and pretested questionnaire was then administered to the patients via face-to-face interview by the investigator.

The first part of the questionnaire dealt with the demographic data including age, sex, marital status, education level, employment status, etc. The second part consisted of details related to prior exposure to anaesthesia and surgery. The third part consisted of 10 pre-validated questions addressing the anxiety factors of patients related to anaesthesia and surgery. The final part consisted of 10 questions addressing various factors causing fear related to the COVID-19 pandemic. All questions were scored on a 1-5 Likert Scale according to the severity of fear ranging from 1 (no fear) to 5 (extreme fear). During data collection, if patients with an extreme degree of stress were identified, they were offered a referral to the counselling centre and helpline numbers. Sample size calculation based on a relative precision of 20%, an expected prevalence of preoperative anxiety of 50%, and a confidence level of 95% gave a required sample of at least 110 patients. The data collected were entered in a spreadsheet, and continuous data and categorical data were recorded into numerical variables and expressed as mean, median, and frequency, respectively.The descriptive analysis of the data was done using SPSS (Statistical Package for Social Sciences) software version 22 (IBM Corp., Armonk, USA).

## Results

A total of 112 patients were enrolled in the study. Baseline characteristics in the study population are shown in Table 1. Out of 112 enrolled in the study, 61 (54.5%) of the patients were females. The mean age of the study participants was 42.3±14.2 years. Most of them were married 96 (85.7%) and resided in urban areas 85 (75.9%). Around 52 (46.4%) graduated and 55 (49.1%) were homemakers. Around 36 (32.1%) of patients have preexisting comorbidity, hypertension being the most common 24 (21.6%).

**Table 1:** Demographic details of study participants

Characteristics	Groups	Number ( N)	Percentage(%)
Age (in years)	<20	04	3.6
	21-30	26	23.2
	31-40	22	19.6
	41-50	31	27.7
	51-60	17	15.2
	>60	12	10.7
Sex	Male	51	45.5
	Female	61	54.5
Marital status	Unmarried	10	8.9
	Married	96	85.7
	Widow/ widower	6	5.4
Education	Illiterate	2	1.8
	Primary	8	7.1
	Middle	0	0
	Secondary	19	17
	Higher	31	27.7
	Graduate and above	52	46.4
Residence	Urban	85	75.9
	Rural	27	24.1
Occupation	Unemployed	3	2.7
	Retired person	7	6.3
	Housewife	55	49.1
	Government Job	10	8.9
	Businessmen/ businesswomen	29	25.9
	Student	8	7.1
Type of family	Joint	27	24.1
	Nuclear	85	75.9
Any existing comorbidity?	Yes	36	32.1
	Diabetes	2	1.8
	Hypertension	24	21.6
	Hypothyroidism	5	4.5
	Heart Disease	1	0.9
	Asthma	1	0.9
	Chronic Obstructive Pulmonary Disease (COPD)	2	1.8
	No	76	68.5

**Figure 1:**
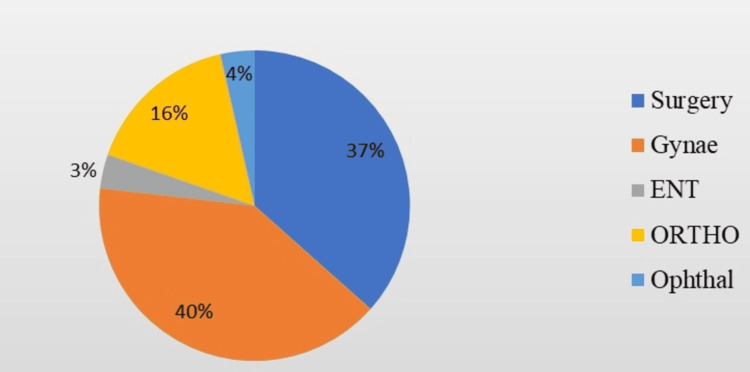
Surgical procedures performed by various departments.

**Figure 2:**
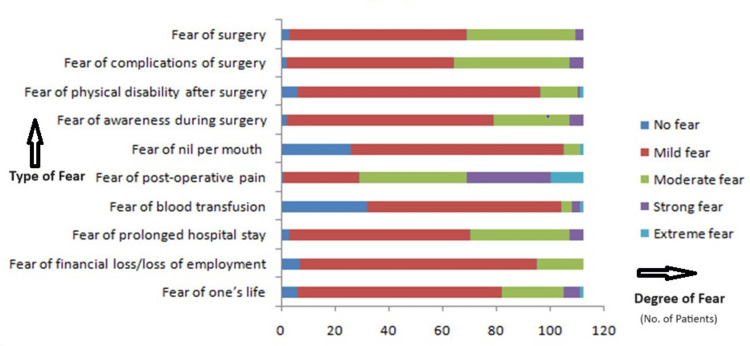
Fear related to surgery and anesthesia.

All patients underwent elective surgery - 112 (100%). The majority - 45 (38.5%) - of the procedures performed during the study period were gynaecological surgeries. Around 65 (58.0%) had undergone minor surgical procedures. Figure 1 shows the surgical procedures performed by various departments. Most of the patients had no prior history of undergoing any surgical procedures - 87 (77.7%). The study assessed various fears related to surgery and anaesthesia, as shown in Figure 2. The most prevalent fears among the participants were fear of postoperative pain - 111 (99.1%), complications of surgery - 110 (98.2%), and fear of surgery itself - 109 (97.3%). The least prevalent fear was the fear of nil per mouth, with 26 (23.2%) reporting no fear and 79 (70.5%) reporting mild fear. The average overall fear scores indicated that most participants experienced mild fear - 71 (62.95%), followed by moderate fear - 25 (22.5%), strong fear - six (5.26%), and extreme fear - two (1.43%). Only eight (7.86%) reported no fear related to surgery and anaesthesia. The results highlight significant anxiety related to various aspects of surgery and anaesthesia among patients during the COVID-19 pandemic. Understanding these fears can help healthcare providers develop targeted interventions to alleviate preoperative anxiety and improve patient outcomes.

**Figure 3:**
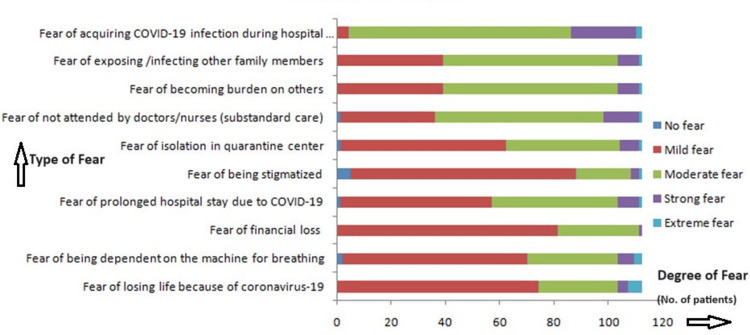
Fear related to COVID-19 pandemic.

The study revealed that all participants - 112 (100%) - experienced some level of COVID-19-related fear, as shown in Figure 3. Predominant concerns included acquiring COVID-19 during a hospital stay [82 (73.2%) moderate fear, 24 (21.4%) strong fear] and the risk of infecting family members [59 (52.7%) moderate fear, 40 (35.7%) strong fear]. Additionally, significant fears were related to financial loss [81 (72.3%) mild fear], prolonged hospital stay [56 (50%) mild fear], and receiving substandard care [62 (55.4%) moderate fear]. Notably, the average overall fear scores indicated moderate to high anxiety levels, underscoring the profound psychological impact of the pandemic on patients awaiting surgery.

## Discussion

The COVID-19 pandemic has emerged as a major global stressor, profoundly affecting individuals worldwide. While the initial focus was on the physical consequences of the infection, it has become clear that the significant psychological impacts of the pandemic also need to be addressed. These psychological consequences may arise from the direct effects of the infection, the restrictive measures imposed to control its spread, or the socio-economic impact of the pandemic. The disruption of healthcare systems worldwide, including the postponement or cancellation of elective surgeries, has further exacerbated these stressors.

This study aimed to assess surgery-related anxiety among patients during the COVID-19 pandemic, focusing on various contributing factors. Our findings indicate significant levels of preoperative anxiety driven by fears related to surgery, anesthesia, and COVID-19 itself. Most participants in our study experienced mild fear (62.95%, 71 out of 112), followed by moderate fear (22.5%, 25 out of 112), with only a small fraction reporting strong (5.26%, six out of 112) or extreme fear (1.43%, two out of 112). Jiwanmall et al. measured anxiety using the Amsterdam Preoperative Anxiety and Information Scale (APAIS), finding significant differences in anxiety scores between cases and controls [9]. The difference in measurement tools and timing of anxiety assessment might account for the variation in reported anxiety severity between the studies.

The present study's data on fears related to surgery and anesthesia among patients highlight significant anxiety levels. All patients reported some degree of fear related to various aspects of surgery and anesthesia. Notably, 58.9% (66 out of 112) of patients reported mild fear, and 35.7% (40 out of 112) reported moderate fear of the surgery itself. These findings are consistent with those of Masood et al., who observed high levels of preoperative anxiety in female patients, with a mean anxiety score of 68.94 for surgery. The fear of complications during surgery was another significant concern, with 55.4% (62 out of 112) of patients experiencing mild fear and 38.4% (43 out of 112) reporting moderate fear. This aligns with Masood et al. study, where fear of postoperative pain and physical disability were prominent anxiety factors [12,13].

The fear of physical disability post-surgery was notably high, with 80.4 (90 out of 112) of patients experiencing mild fear. In comparison, Mertens et al. found that anxiety about the health and safety of loved ones was a substantial predictor of overall fear, suggesting that patients' concerns extend beyond their health to potential impacts on their quality of life and family responsibilities [7]. Our study also found that 68.8% (77 out of 112) of patients feared awareness during surgery, a specific and intense fear reported in 25% (28 out of 112) of patients as moderate fear. This concern is also reflected in Masood et al. findings, where the fear of being awake or aware during surgery was significantly higher among female patients [12].

Interestingly, 70.5% (79 out of 112)of patients reported mild fear of fasting (nil per mouth) before surgery, and 27.7% (31 out of 112) had strong fear of postoperative pain, highlighting the anticipatory anxiety about discomfort and pain management. The fear of blood transfusion was present in 64.3% (72 out of 112) of patients, reflecting concerns about the invasiveness and potential complications of surgical procedures.

Furthermore, the fear of prolonged hospital stay was significant, with 59.8% (67 out of 112) of patients reporting mild fear and 33% (37 out of 112) reporting moderate fear. This concern is exacerbated by the COVID-19 pandemic, as highlighted in Tilmans et al.'s study, where preoperative screening did not completely alleviate patients' anxiety about hospital-related risks [14]. Financial loss or loss of employment was a mild fear for 78.6% (88 out of 112) of patients, indicating the socioeconomic implications of surgery during the pandemic.

The fear of life-threatening outcomes was present in 67.9% (76 out of 112) of patients, with 20.5% (23 out of 112) experiencing moderate fear. This aligns with the general anxiety about health and mortality observed in pandemic-related studies, such as those by Rentero et al., who reported heightened anxiety and psychosis related to COVID-19 [15,16].

The present study found that the most prevalent fears among participants were related to the complications of surgery (98.2%, 110 out of 112) and postoperative pain (99.1%, 111 out of 112). Additionally, COVID-19-specific fears were significant, with 96.4% (108 out of 112) fearing acquiring the infection during their hospital stay and 89.3% (100 out of 112) fearing infecting family members. These high levels of fear reflect the heightened anxiety due to the dual stressors of surgery and the pandemic. Similarly, Masood et al. reported high levels of anxiety related to postoperative pain (93.8%, 75 out of 80 in females), fear of one’s life (71.3%, 57 out of 80 in females), and concerns about family (92.5%, 74 out of 80 in females). Notably, Masood et al. found that change of environment (80.0%, 64 out of 80), waiting for operation (77.5%, 62 out of 80 ), and awareness during surgery (58.8%, 47 out of 80) were significant factors contributing to increased anxiety in females compared to males [12]. Mertens et al. focused on fears specifically related to COVID-19, finding that the strongest predictor of fear was the risk of infection for loved ones [7]. Other significant predictors included looking up additional information through various media sources, which was associated with increased fear of the coronavirus. These results align with our findings where the fear of exposing family members and the impact of the media on anxiety levels were prominent.

Comparing our findings with pre-pandemic data underscores the increased anxiety levels due to the pandemic. While fear of postoperative pain and complications has always been prevalent, the additional fear of contracting COVID-19 during hospital visits has notably increased overall anxiety levels. This highlights the profound impact of the pandemic on patients' psychological states.

This study has several strengths, including a robust sample size and a comprehensive assessment of anxiety factors. However, it also has limitations, such as its cross-sectional design and potential self-reporting biases. Despite these limitations, the findings provide significant insights into the impact of the COVID-19 pandemic on preoperative anxiety. Future research should explore the long-term impact of pandemic-related anxiety on surgical outcomes through longitudinal studies. Additionally, investigating the effectiveness of various interventions to reduce preoperative anxiety in similar contexts would provide valuable insights for healthcare providers.

## Conclusions

The COVID-19 pandemic has significantly heightened preoperative anxiety among patients scheduled for surgery. Addressing both surgical and pandemic-related anxieties is crucial for improving patient outcomes. Healthcare providers should consider implementing psychological support programs and tailored interventions to mitigate these anxieties. Understanding the multifaceted nature of preoperative anxiety during the pandemic can help enhance patient care.
